# Regulation of Memory Function by Feeding-Relevant Biological Systems: Following the Breadcrumbs to the Hippocampus

**DOI:** 10.3389/fnmol.2019.00101

**Published:** 2019-04-18

**Authors:** Andrea N. Suarez, Emily E. Noble, Scott E. Kanoski

**Affiliations:** Human and Evolutionary Biology Section, Department of Biological Sciences, University of Southern California, Los Angeles, CA, United States

**Keywords:** hippocampus, memory, obesity, vagus nerve, GLP-1, learning, plasticity, neurogenesis

## Abstract

The hippocampus (HPC) controls fundamental learning and memory processes, including memory for visuospatial navigation (spatial memory) and flexible memory for facts and autobiographical events (declarative memory). Emerging evidence reveals that hippocampal-dependent memory function is regulated by various peripheral biological systems that are traditionally known for their roles in appetite and body weight regulation. Here, we argue that these effects are consistent with a framework that it is evolutionarily advantageous to encode and recall critical features surrounding feeding behavior, including the spatial location of a food source, social factors, post-absorptive processing, and other episodic elements of a meal. We review evidence that gut-to-brain communication from the vagus nerve and from feeding-relevant endocrine systems, including ghrelin, insulin, leptin, and glucagon-like peptide-1 (GLP-1), promote hippocampal-dependent spatial and declarative memory *via* neurotrophic and neurogenic mechanisms. The collective literature reviewed herein supports a model in which various stages of feeding behavior and hippocampal-dependent memory function are closely linked.

## Introduction

“*Ponder well on this point: the pleasant hours of our life are all connected by a more or less tangible link with some memory of the table*.”

*—Charles Monselet*.

In most corners of the globe food is readily available with minimal effort required for foraging. This luxury of the modern environment was not enjoyed throughout human evolution, as food in nature was often irregularly available to our ancestors and required substantial labor and resources to obtain. A survival advantage common to early humans and lower-order mammals is to accurately remember the physical location of a food source, and to then efficiently navigate back to a shelter/dwelling. Thus, it follows that the biological systems that regulate feeding behavior may share common origins with those involved in learning about one’s external environment. Here, we present a framework that the ability to remember the location and features of environmental locations (visuospatial and contextual memory, respectively) is intimately linked with endocrine and neural pathways that also regulate appetite and feeding behavior.

In addition to external spatial and contextual cues, learning about other aspects of feeding behavior also provides a survival advantage. For example, in humans and in lower-order mammals (including rodents), social cues strongly influence both food choice and amount consumed, in part, by conveying that a particular food is safe and/or nutritive (de Castro et al., 1990; Levitsky, 2005; Herman and Higgs, 2015). Further, memory of the physical location of a food source is modulated by temporal factors, including longer-term seasonal changes for vegetation and shorter-term diurnal fluctuations for predator-prey dynamics. Remembering these and other aspects of the entire “feeding episode” are useful to more efficiently guide future foraging and feeding behavior. Thus, biological systems that fluctuate around and influence feeding behavior may also promote a hippocampal-dependent memory process known as declarative memory, which is the flexible memory for facts and episodic events (Eichenbaum and Cohen, 2014). We note, however, that declarative memory and visuospatial memory are not necessarily two distinct processes, but rather, may be two manifestations of the same (or similar) fundamental memory process that is mediated by hippocampal neurons (Buzsáki and Moser, 2013; Milivojevic and Doeller, 2013; Eichenbaum and Cohen, 2014).

During the preprandial (before a meal), prandial, and postprandial stages of feeding behavior, various endocrine, neuropeptidergic, and neural signals are released to regulate appetite, meal size, and the inter-meal interval. Emerging evidence reveals that in addition to regulating feeding behavior, these energy status-related biological systems also play a critical role in learning and memory function, particularly with regards to remembering feeding-relevant episodic experiences and visuospatial information such as recalling when, how, and where food was obtained and consumed. Here, we review evidence that these energy status-related biological signals converge with external sensory-related cues in the hippocampus (HPC), a brain region that is most famously linked with learning and memory and more recently with the higher-order controls of feeding behavior (Davidson et al., 2007; Parent et al., 2014; Kanoski and Grill, 2017). Indeed, HPC neurons play an important role in processing relative information about one’s external environment and spatial orientation (Morris et al., 1982; Burgess et al., 2002). The HPC is also required for flexible memory for autobiographical events (“episodic memory,” a component of declarative memory; Tulving and Markowitsch, 1998; Eichenbaum and Cohen, 2014; Parent, 2016).

Consistent with a role for HPC neurons in regulating spatial and declarative memory processes relevant to foraging and feeding, selective HPC lesions impair both meal-related spatial and episodic memory used to relocate previously stored food sources in scrub jays (Clayton and Dickinson, 1998). Similarly, intact rhesus monkeys preferentially return to a foraging site where food was previously obtained in a matching-to-location task, whereas monkeys with HPC lesions do not show this learned matching tendency (Hampton et al., 2004). While selective targeted lesions to the HPC is not a feasible experimental approach in humans for obvious reasons, amnesic humans with bilateral nonselective damage to the HPC and surrounding medial temporal lobe structures (including the amygdala) will eat a full second meal that is offered immediately after consuming a meal with minimal change in self-reported hunger/satiety ratings (Hebben et al., 1985). Analogous findings have been observed in rodents in which reversible postprandial inactivation of either dorsal (dHPC) or ventral (vHPC) hippocampus using either muscimol infusions (Henderson et al., 2013; Hannapel et al., 2017) or optogenetic inhibition (Hannapel et al., 2019) decreases latency to initiate the subsequent meal and increases subsequent intake, results that are hypothesized to be based on disrupted meal-related episodic memory consolidation (Parent, 2016). These findings further indicate that both the dorsal and ventral HPC subregions are involved in meal-related memory processing, which is notable in light data suggesting that these subregions may be functionally distinct with regards to spatial memory (dHPC) and emotional-related memory processing (vHPC; see Moser and Moser, 1998; Fanselow and Dong, 2010; Kanoski and Grill, 2017; for further review on dorsal vs. ventral dissociation of function). Also of note from Parent and colleagues’ work is that reversible optogenetic inactivation of either dHPC or vHPC neurons reduces the latency to initiate a subsequent meal following nonnutritive saccharine consumption (Hannapel et al., 2019), suggesting that HPC control of meal-related episodic memory may be based, in part, on hedonic orosensory properties independent of post-ingestive caloric consequences.

These findings indicate that a critical bridge between previous eating episodes, interoceptive energy status cues, and ongoing feeding behavior resides within the HPC. Consistent with this framework, selective lesions to the HPC in rats yields increased food intake and body weight gain (Davidson et al., 2010), as well as increased meal frequency (Clifton et al., 1998). These results were obtained in rats free feeding in the home cage, and therefore are unlikely to be based on deficits in visuospatial memory and foraging, but rather, may be based on impaired episodic memory of recent eating occasions (e.g., see Parent et al., 2014).

In this review article, we discuss the common biological mechanisms through which feeding behavior and HPC-dependent memory function are closely linked. More specifically, we focus primarily on how peripherally-derived feeding-relevant signals that are released before, during, and after a meal exert their action directly on their respective targets in HPC neurons to influence both feeding behavior and memory function, particularly mnemonic processes related to autobiographical events, foraging, and food location.

## Ghrelin

### System Overview

Ghrelin, often referred to as the “hunger hormone,” is a 28-amino acid peptide hormone produced and secreted by P/D1 cells in the fundus of the stomach (Kojima et al., 1999; Date et al., 2000; Dornonville de la Cour et al., 2001). Ghrelin binds to and activates its seven transmembrane G protein couple receptor, the type 1a growth hormone secretagogue receptor (GHSR1a, aka “ghrelin receptor”; Howard et al., 1996; Sun et al., 2004). The pre-pro ghrelin precursor protein is first cleaved into two peptides, obestatin and des-acyl ghrelin (Gualillo et al., 2006). Des-acyl ghrelin is present in the stomach and bloodstream and is the inactive form of ghrelin, as it does not engage GHSR1a signaling at physiological concentrations (Hosoda et al., 2000; Tong et al., 2013). During times of energy insufficiency, the ghrelin O-acyltransferase (GOAT) enzyme is upregulated and converts des-acyl into its active acyl form by adding an acyl chain on Ser3 residue of ghrelin (Gahete et al., 2010; Zhao et al., 2010). Following this post-translational modification, acyl ghrelin can then access the active GHSR1a binding sites to augment both appetite and food intake (Yang et al., 2008). Consistent with ghrelin’s orexigenic effects, ghrelin levels are elevated during energy restriction, peak preprandially (Wren et al., 2001a; Drazen et al., 2006; Blum et al., 2009; Davis et al., 2011), and rapidly decrease in response to eating (Ariyasu et al., 2001; Cummings et al., 2001; Nass et al., 2008).

Early research on ghrelin signaling in the brain largely focused on regions classically associated with energy homeostasis [e.g., arcuate nucleus of the hypothalamus (ARH), nucleus tractus solitarius (NTS) in the caudal brainstem] (Wren et al., 2001b; Faulconbridge et al., 2003). However, GHSR1as are also expressed in “higher-order” (limbic, cortical) brain regions involved in memory and cognition, including the HPC (Guan et al., 1997; Zigman et al., 2006). More specifically, GHSR1a is robustly expressed in the dentate gyrus (DG), CA1, CA2, and CA3 regions of the HPC, particularly in the vHPC (Guan et al., 1997; Diano et al., 2006; Zigman et al., 2006; Mani et al., 2014; Hsu et al., 2015b). While the HPC is protected by the blood-brain barrier (BBB), radiolabeled ghrelin is present in the mouse HPC following peripheral administration (Harrold et al., 2008). Bioactive ghrelin may reach HPC neurons either through acyl ghrelin BBB saturable transport, or through des-acyl ghrelin crossing the BBB from blood to brain uni-directionally and conversion to acyl ghrelin within the central nerve system (CNS) *via* GOAT transcripts expressed in the brain (for review, see Edwards and Abizaid, 2017), as minimal evidence supports the idea that ghrelin is produced in the brain (Ferrini et al., 2009).

### Memory Function

Research on ghrelin has predominantly focused on its role in appetite and food intake. Ghrelin signaling also influences memory and cognition, and here we consider that these lesser studied effects are very much connected with ghrelin’s role in appetite. Early evidence for ghrelin’s role in modulating cognitive function comes from Carlini et al. (2002), who demonstrated that administration of acyl ghrelin in the cerebral ventricles (Carlini et al., 2002) or the HPC directly (Carlini et al., 2004) immediately after training improved memory retention in a step-down inhibitory avoidance assay in a dose-dependent manner in rats, indicating a stimulatory effect of ghrelin on memory consolidation for contextual episodic memory. Further work from this group revealed that intra-hippocampal ghrelin administration before training sessions improved memory for the contextual location of aversive reinforcement (Carlini et al., 2010). Diano et al. (2006) extended this work and demonstrated that subcutaneous ghrelin administration in rats improves performance in a spontaneous alternation plus-maze task, whereas intracerebroventricular (ICV) ghrelin administration following training enhanced retention performance in T-maze foot shock avoidance and step-down passive avoidance tasks in mice.

While these pharmacological findings suggest a role for ghrelin in memory consolidation, it is important to consider whether endogenous ghrelin plays a physiological role in memory. Indeed, ghrelin KO mice (Diano et al., 2006) are impaired in a novel object recognition (NOR) task, and these deficits are rescued following subcutaneous ghrelin replacement. Recently the endogenous ghrelin antagonist, liver-expressed antimicrobial peptide 2 (LEAP2), was identified. Not surprisingly, LEAP2 administration reduces food intake, blocks fasting-induced growth hormone secretion, and impairs the maintenance of glucose levels during chronic caloric restriction, however, to our knowledge its role in memory function and neuronal plasticity has yet to be examined (Ge et al., 2018).

The rodent model work described above uses memory tasks that involve learning about external cues and/or episodic memory based on either passive or aversive reinforcement. These fundamental learning processes may be advantageous to inform about future feeding behavior, however, more direct evidence that ghrelin promotes feeding-relevant memory comes from studies that utilize food reinforcement. Considering that ghrelin levels peak before a meal, ghrelin may facilitate food seeking by enhancing HPC-dependent spatial and contextual memory to remember the physical location of food sources, as well as other features (e.g., social factors) that comprise episodic memory relating to appetitive and consummatory behavior. Indeed, wild-type mice treated with a GHSR antagonist and GHSR-null mice fail to show conditioned place preference (CPP) to a high fat diet (HFD; Perello et al., 2010; Chuang et al., 2011; Disse et al., 2011), demonstrating that ghrelin plays a role in enhancing memory for the location of reward-based food intake. Consistent with this framework, wildtype mice exhibit Pavlovian cue-induced hyperphagia following extensive cue-food conditioning (i.e., “cue-potentiated feeding”), whereas GHSR1a-null mice do not (Walker et al., 2012). This deficit may be based, in part, on the loss of GHSR1a in the ventral subregion of the HPC (vHPC), as vHPC ghrelin administration increases food-motivated behaviors for sucrose reinforcement and increases initiation of meals in response to external food-related cues in rats (Kanoski et al., 2013). Moreover, vHPC GHSR1a blockade prior to chow access reduces food intake in meal-entrained rats that had previously learned to consume all of their daily calories in a 4 h period, yet has no effect on intake in rats that were equally food restricted but were not previously meal-entrained (Hsu et al., 2015b). Consistent with these pharmacological findings, GHSR1a-null mice lack food anticipatory activity to habituated feeding responses (Davis et al., 2011).

In addition to promoting appetitive-related memory based on external discrete cues, contextual cues, and temporal scheduled feeding cues, additional rodent model work reveals that ghrelin signaling in HPC promotes social-based memory related to feeding. We recently examined the effects of RNA interference-mediated knockdown (KD) of ventral CA1 GHSR1a [*via* an adeno-associated virus (AAV) expressing short hairpin RNAs targeting GHSR1a] on learning the olfactory-based social transmission of food preference task (STFP; Hsu et al., 2018). In this task, “observers” rats learn to prefer a flavor of chow based on a brief social interaction with another “demonstrator” rat that had recently consumed the flavored chow. The “transmission” of food preference in observer rats, demonstrated subsequently as a preference for the demonstrator-paired flavored chow vs. a novel flavored chow, is based on exposure to olfactory cues from the breath of the demonstrator rat during the social interaction (Countryman et al., 2005). Rats that received a control AAV in the vHPC (with scrambled sequence for the short hairpin RNAs) preferred the demonstrator-paired flavored chow vs. the novel flavored chow, whereas rats with vHPC GHSR-1a KD were impaired in learning the STFP task. Importantly, a non-social olfactory transmission of food preference control task was performed in this study to determine whether the deficits found in vHPC GHSR1a KD is specific to olfactory processing of social cues (Hsu et al., 2018). This task replicates the olfactory components of the STFP task but excludes social interaction with a demonstrator rat in the social interaction arena. Results from this control procedure show that both vHPC GHSR1a KD and control animals preferred the flavored chow that was paired with the bedding, indicating vHPC GHSR1a KD selectively impairs learned, social transmission of food preference without deficits in olfactory processing or habituation learning.

Evidence from humans also supports the idea that ghrelin signaling enhances attention to external food-related cues, and this enhanced attention may serve to promote the acquisition and consolidation of food-related memories *via* action in the HPC. For example, ghrelin administration to healthy volunteers during functional magnetic resonance imaging increases cerebral blood flow (CBF) response to food cues, and in the HPC and amygdala, this effect is specific to food cues with no change in CBF in response to scenery pictures (Malik et al., 2008). A more recent article expanded this work by showing that the ability of hyperghrelinemia (either endogenous or exogenous-based) to enhance food-cue induced increased CBF, including in the HPC, is not explicable by consistent changes in other metabolic markers that vary with energy status, including glucose, insulin, peptide YY, and glucagon-like peptide-1 (GLP-1; Goldstone et al., 2014). Moreover, intravenous ghrelin administration in healthy humans enhances the formation of food-related cue-reward associations by increasing HPC signaling to ventral striatum (Han et al., 2018), results consistent with a previously discussed study in rodents demonstrating an important role for vHPC ghrelin signaling in external social-based food cues (Hsu et al., 2018).

### Neurobiological Mechanisms

Ghrelin enhances memory function, in part, by promoting adult hippocampal neurogenesis and synaptic plasticity. Ghrelin administered peripherally induces proliferation and differentiation of adult progenitor cells in the DG (Moon et al., 2009; Zhao et al., 2014), whereas immunoneutralization of ghrelin in the DG reduces these effects (Moon et al., 2009). Furthermore, ghrelin regulates morphological changes in HPC neurons as ghrelin receptor KO rodent models are associated with reduced HPC spine density (Cahill et al., 2014), whereas reduced HPC spine density is rescued following peripheral ghrelin administration in ghrelin deficient rodents (Diano et al., 2006). *In vitro* ghrelin administration also enhances long-term potentiation (LTP) in HPC slices (Diano et al., 2006), whereas *in vivo* ghrelin administration directly in the DG enhances synaptic plasticity [e.g., long-lasting potentiation of excitatory postsynaptic potentials (EPSPs)] and improves spatial memory in the Morris water maze task *via* activation of the PI3K signaling pathway (Chen et al., 2011). Collectively these findings show that ghrelin enhances neurogenesis and neural plasticity in the HPC and that these processes are feasible neurobiological mechanisms through which ghrelin promotes memory processes.

### Interactions With Diet and Energy Status

Considering that plasma ghrelin concentrations increase during calorie restriction (Yang et al., 2007), and that calorie restriction increases synaptic plasticity (Fontán-Lozano et al., 2007) as well as ghrelin BBB transport (Harrold et al., 2008; Schaeffer et al., 2013), it is likely that ghrelin’s role in enhancing memory function is very much dependent on energy status. Consistent with this notion, overnight calorie restriction (or intraperitoneal acyl-ghrelin administration) increased levels of early growth response-1 (Egr-1; a neurogenic transcription factor) in the DG, and 2 weeks on a calorie restriction diet improved contextual fear memory and increased neurogenesis in the DG of wild type but not GHSR deficient mice (Hornsby et al., 2016). Additionally, when ghrelin KO and wild-type mice were maintained on either dietary restriction or *ad libitum* feeding for 3 months, the ghrelin KO mice on an *ad lib* diet demonstrated reduced neurogenesis, whereas dietary restriction increased the survival of newborn cells in wild type, but not ghrelin knock out mice (Kim et al., 2015). These studies indicate that endogenous GHSR and ghrelin signaling during calorie restriction play a critical role in enhancing HPC neurogenesis and memory function induced by energy deficits.

On the other side of the energy balance scale, obese humans (English et al., 2002; Yildiz et al., 2004) and diet-induced obese rodents (Perreault et al., 2004; Williams et al., 2006; Uchida et al., 2014) exhibit attenuated ghrelin secretion and reduced circulating plasma ghrelin levels. Obese mice also demonstrate deficits in ghrelin BBB permeability (Banks et al., 2008), as well as reduced hyperphagia (Perreault et al., 2004) and hypothalamic arcuate NPY/AgRP mRNA expression (Briggs et al., 2010) in response to peripheral administration of ghrelin. In humans, lean individuals homozygous for the obesity risk-associated fat mass and obesity-related (FTO) gene allele demonstrate attenuated postprandial reduction of circulating levels of acyl-ghrelin, as well as attenuated difference in BOLD responsiveness to high-calorie vs. low-calorie food images in homeostatic and hedonic brain regions in the fed vs. fasted state (Karra et al., 2013). Similarly, studies have identified CNS “resistance” to ghrelin-induced food reward-associated behaviors in DIO mice. For example, peripheral ghrelin administration enhances operant progressive ratio (PR) responding for sucrose reward (Finger et al., 2012) and induces CPP for palatable food availability in normal but not DIO mice (Lockie et al., 2015). Whether DIO impairs feeding-related memory function *via* vHPC GHSR1A signaling remains to be determined, but HFD-induced ghrelin resistance has been identified at the intracellular signaling level in HPC neurons, as ICV ghrelin administration increases vHPC PI3K and Akt signaling in chow-fed, but not HFD-rats (Kanoski et al., 2013).

### Concluding Framework

**Figure 1:**
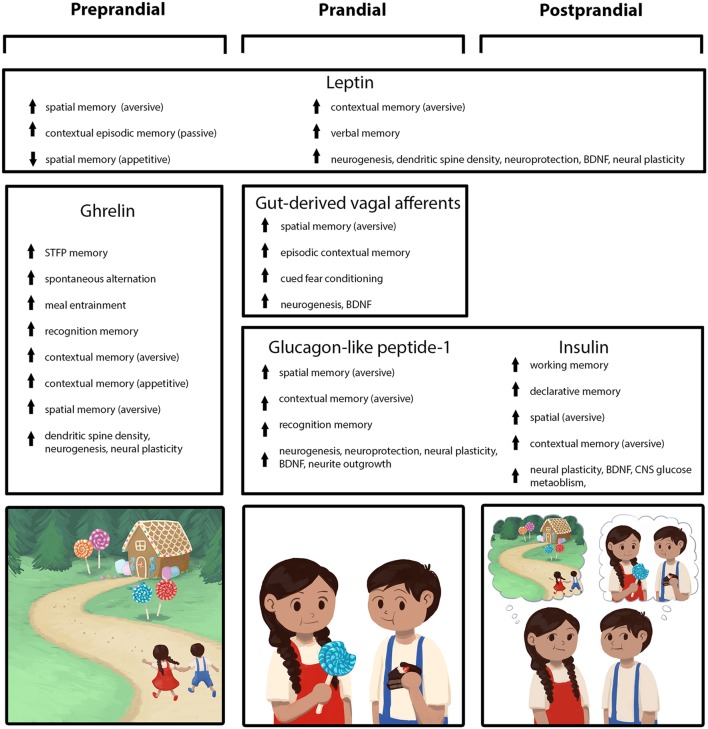
Peripherally-derived feeding-relevant signals occurring during the preprandial (left column), prandial (center column), and postprandial (right column) stages of feeding converge with neural processing in the hippocampus (HPC) to promote learning and memory for various elements of a feeding episode. These mnemonic episodic elements include the spatial location of a food source, as well as the temporal, nutritive, and social aspects of a meal (mnemonic elements depicted in the cartoon across the bottom row). During the preprandial/appetitive stage, elevated ghrelin signaling promotes HPC-dependent spatial and episodic memory formation related to food procurement *via* GHSR action in HPC neurons. In the prandial stage, within-meal gut-derived vagal sensory signaling enhances HPC-dependent memory for visuospatial and external contextual features related to a feeding episode *via* an ascending multisynaptic hindbrain-septal-HPC pathway. In addition, endocrine signals (glucagon-like peptide-1, GLP-1, insulin) are released prandially and immediately postprandially, which each independently contribute to meal-related episodic mnemonic elements (e.g., memory of the nutritive quality of a meal, food location) *via* direct action on HPC neurons, as well as indirectly through metabolic pathways. The adipokine leptin is presented as a signal that influences HPC-dependent memory (*via* direct action on HPC neurons) across all feeding stages, potentially *via* a modulatory mechanism such that optimal leptin levels associated with healthy energy status promote food-related memory.

Overall these findings reveal that ghrelin signaling in the HPC is physiologically relevant for conditioned aspects of appetitive behavior. In addition to promoting HPC-dependent memory based on external spatial and contextual cues in tasks that involve passive or aversive reinforcement, emerging research detailed above shows that ghrelin promotes HPC-dependent memory directly related to feeding behavior, including learning about discreet external cues, contextual external cues, temporal entrainment cues, and social cues that inform about feeding. When in a state of chronic or acute energy deficit, ghrelin signaling may be adaptive to memory formation and retrieval of a food source location and other episodic elements of feeding-related events (Figure 1). On the other hand, the capacity of ghrelin signaling to promote food intake, conditioned appetitive behaviors, and intracellular signaling cascades in HPC neurons may be blunted in obesity and with HFD maintenance. Given that ghrelin levels rapidly decrease following the onset of eating, it follows that additional feeding-relevant systems that mediate prandial, postprandial, and overall energy status are also likely to interact with HPC-dependent memory function. These systems are discussed below.

## Insulin

### System Overview

Insulin is a peptide hormone produced by pancreatic β cells in the islets of Langerhans that plays an important role in nutrient metabolism and energy homeostasis. A major function of insulin in the periphery is to facilitate postprandial storage of nutrients, thereby maintaining nutrient homeostasis in the blood following a meal. The most well-characterized of the insulin secretion pathways is metabolic, whereby intracellular catabolism of nutrients by the β cell triggers a rise in intracellular calcium and insulin secretion (for review, see Skelin Klemen et al., 2017). While glucose is the nutrient most commonly associated with insulin secretion, foods that are low in glucose but high in proteins and fats also raise plasma insulin levels (Holt et al., 1997). In addition to this endocrine metabolic pathway, a neural pathway *via* vagal inputs to the pancreas mediates a cephalic insulin response, which elevates insulin secretion within the first few minutes of initiating a meal (triggered, in part, by oral nutrient detection; Berthoud et al., 1981; Powley, 2000; Ahrén and Holst, 2001). Finally, a humoral pathway for insulin secretion exists involving the incretin hormones GLP-1 and glucose-dependent insulinotropic polypeptide (GIP), which act in concert with rising glucose levels to stimulate insulin synthesis and secretion *via* an adenylate cyclase mediate pathway (Cernea and Raz, 2011).

Insulin acts on the insulin receptor (IR), which belongs to the receptor tyrosine kinase superfamily (De Meyts, 2000). Insulin binding to IR in muscle or adipose tissue promotes glucose uptake into the cell, as well as glycogen, fat, and protein synthesis (Tatulian, 2015). In addition to being expressed in peripheral tissues capable of storing nutrients, IR is expressed in the brain, where it plays a role in both peripheral energy metabolism and reducing food intake (Woods et al., 1996; Bruning et al., 2000; Schwartz et al., 2000; Obici et al., 2002a,b). High levels of IR expression in the brain are found in the olfactory bulb, cerebral cortex, hypothalamus, cerebellum, cortex, and HPC, with robust expression in the pyramidal region of the CA1, CA3, and granule layer of the DG (Unger et al., 1989; Marks et al., 1990; Schulingkamp et al., 2000). The major source of insulin in the brain is believed to come either predominantly or exclusively from the periphery *via* a BBB saturable transporter system (Baura et al., 1993; Banks, 2004; Ferrario and Reagan, 2018). The process through which insulin is transported across the BBB was recently elucidated, in part, by Gray et al. (2017), who showed that insulin enters the brain *via* IR-mediated brain endothelial cell transcytosis, a vesicle-mediated process that does not require downstream signaling of the classic IR intracellular PI3K signaling cascade. Insulin uptake across the BBB is not uniform across brain regions, however, and some regions appear to be more permeable for insulin transport than others. Notably, along with the pons, medulla, and the hypothalamus, the HPC is one of the regions with the highest uptake for insulin from the blood (Banks and Kastin, 1998). Thus, the HPC is a region containing both high levels of IR and for which the BBB is particularly permeable for insulin, suggesting that endogenous peripheral changes in insulin levels influence hippocampal function.

### Memory Function

Early evidence for insulin’s involvement in memory was reported by Strong et al. (1990) who discovered that peripheral injections of insulin completely reversed deficits in working memory attributable to the ischemic stroke. Subsequently, Craft et al. (1996) showed that insulin alone, independent of the consequential lowering of blood glucose, is sufficient to significantly improve hippocampal-dependent declarative memory function in human subjects with mild symptoms of dementia. In healthy human subjects, 8 weeks of intranasal insulin treatment (4×/day) significantly improves hippocampal-dependent declarative memory (Benedict et al., 2004). Additional evidence for a role of insulin in supporting cognitive functioning comes from rodent models of diabetes, in which insulin secretion is reduced. For example, rats treated with intravenous injection of streptozotocin (STZ), which kills insulin-secreting pancreatic β cells, have impaired hippocampal-dependent place/location learning and reduced synaptic plasticity (Biessels et al., 1996). Rats treated similarly with STZ also have impaired short-term spatial memory, a deficit that is restored by peripheral treatment with insulin (Kumar et al., 2011).

In addition to impaired peripheral and central insulin signaling seen in diabetes models, impaired memory function in Alzheimer’s disease (AD) is associated with disrupted brain IR signaling. In fact, it has been suggested that AD be considered as “diabetes type 3” (Rivera et al., 2005; Steen et al., 2005; de la Monte and Wands, 2008). In humans, intranasal treatment with insulin has been shown to improve delayed memory recall associated with both mild cognitive impairment (MCI) and mild-to-moderate AD (Reger et al., 2008; Craft et al., 2012, 2017). Extracellular deposits of amyloid β and neuronal loss in the HPC are hallmarks of AD, as is impairment in hippocampal-dependent memory function. In rats, ICV injections of STZ, an established model for AD, impairs spatial and working memory and reduces IR signaling molecules, effects that are normalized by intranasal insulin treatment (Rajasekar et al., 2017; Rostami et al., 2017). There is also evidence to support a role for IR signaling in hippocampal-dependent learning and memory performance in the absence of cognitive deficit and/or dementia. For example, insulin injected into the dHPC enhances spatial working memory performance in rats in the spontaneous alternation task (McNay et al., 2010). Similarly, in healthy rats, bilateral dHPC injections of insulin transiently enhance long-term memory in contextual fear conditioning and inhibitory avoidance tasks (Stern et al., 2014) and insulin injected into the CA1 region of the dHPC immediately post-training enhances memory performance in an inhibitory avoidance task (Babri et al., 2007).

In addition to pharmacologically injected insulin, endogenous IR signaling in the HPC is critical for learning and memory function in healthy lab rats. For example, blockade of endogenous insulin in the HPC *via* microinjection of a small, anti-insulin, antibody-like protein, impairs spatial working memory performance in the spontaneous alternation task (McNay et al., 2010) and a 70% KD of dorsal HPC (dHPC) IR impairs HPC-dependent long-term spatial memory without affecting energy balance or peripheral glucose metabolism (Grillo et al., 2015). Consistent with these effects, learning *per se* appears to augment IR expression in the HPC. For example, spatial learning in the Morris water maze is associated with increased IR expression in the CA1 pyramidal region of HPC, as well as enrichment of the receptor in nuclear and dendritic compartments (Zhao et al., 1999). Together, the literature strongly supports a role for IR signaling in HPC-dependent learning and memory function, and further suggests that memory function can be enhanced with exogenous insulin treatment under both pathological and healthy conditions.

### Neurobiological Mechanisms

Whether mechanisms for central IR impairment are similar to peripheral IR resistance is unclear. One major difference between central and peripheral IR function is that a major function of insulin in the periphery is to enhance GLUT4 migration and glucose uptake, whereas glucose uptake in the brain is thought to be largely insulin independent (Tatulian, 2015). However, hippocampal neurons contain GLUT4 transporters and insulin enhances GLUT4 migration to the membrane of hippocampal neurons *via* a PI3K mediated pathway (Reagan, 2005; Grillo et al., 2009). In rats, intrahippocampal injections of insulin increase local glycolytic activity and enhances spatial working memory performance in the spontaneous alternation task *via* a PI3K-mediated pathway (McNay et al., 2010), whereas blocking GLUT4 mediated glucose uptake prevents performance improvements in the task by administration of memory-enhancing doses of insulin to the HPC (Pearson-Leary et al., 2018). Collectively these findings suggest that insulin’s memory-enhancing effects require increased glucose utilization. Indeed, spatial memory is limited by glucose availability and utilization in the HPC (McNay et al., 2000). Thus, the action of insulin in mediating neuronal glucose uptake may, in part, contribute to insulin’s memory enhancing effects.

In addition to improving glucose uptake, at the neuronal level, mechanisms for how central insulin may improve memory function include increasing synaptic plasticity. For example, IR signaling promotes synaptic plasticity (e.g., LTP) in hippocampal neurons, a physiological process widely considered to play a critical role in memory (Lee and Silva, 2009; Lisman et al., 2018). On the other hand, genetically knocking down dHPC IR impairs hippocampal-dependent memory and blocks LTP in the CA1 and DG (Grillo et al., 2015). Related to IR-mediated increases in neuronal plasticity, insulin increases the expression of the growth and plasticity factor brain-derived neurotrophic factor (BDNF) and its receptor TrkB in the HPC (Haas et al., 2016), an effect that is abolished in aged rats. The accumulation of amyloid β in the HPC reduces BDNF function, cell membrane IRs, and IR pathway mediated signaling (De Felice et al., 2009, 2014; Takach et al., 2015). Moreover, the function of BDNF is restored by activating the downstream mediators of IR signaling, suggesting that AB may reduce neuronal plasticity in part by interfering with IR function (Takach et al., 2015). Thus, overall the mechanisms through which central IR signaling may enhance HPC functioning include improved glucose utilization and enhanced neuronal plasticity *via* neurotrophic pathways.

### Interactions With Diet and Energy Status

Central IR function appears to be potently regulated by dietary and metabolic factors. For example, insulin resistance in the HPC is observed in the obese Zucker fa/fa rat model (Špolcová et al., 2014). Similarly, DIO rodents are less responsive to the memory enhancing effects of intrahippocampal insulin (McNay et al., 2010). In females, estrogen treatment normalizes both the peripheral and central IR sensitivity due to ovariectomy in rats fed standard chow, however, peripheral but not central IR sensitivity is restored by estrogen treatment in ovariectomized rats fed a HFD (Pratchayasakul et al., 2014). Even short-term (7 days) feeding of a diet high in saturated fat and fructose is associated with reduced hippocampal IR signaling (Calvo-Ochoa et al., 2014). Similarly, a diet high in fructose (without elevated fat) reduces IR phosphorylation in hippocampal neurons and activation of the downstream signaling molecules IR Substrate-1 (Agrawal et al., 2016), AKT, and PI3K (Wu et al., 2015), outcomes also associated with impaired spatial memory (Wu et al., 2015; Agrawal et al., 2016).

### Concluding Framework

Given that insulin secretion is triggered by immediate nutrient availability, one can speculate that it would be advantageous from an evolutionary perspective to have enhanced spatial and contextual memory during the prandial state in order to remember the location of the food supply, as well as to mnemonically encode additional episodic information that facilitated feeding (Figure 1). Indeed, insulin is an ideal candidate to enhance such memories surrounding the prandial period, as its release occurs at the onset, during, and after a meal. One possible explanation for why memory function is enhanced by insulin and other endocrine factors that are triggered during an ongoing meal is that the memory of the meal-related episodic event may be important for influencing the timing of and amount consumed in a subsequent meal in order to maintain optimal energy balance. Similarly, it is advantageous for animals to be able to encode the memory of the nutritive quality of a meal to better guide future foraging behavior. These memory-promoting effects of insulin appear to mirror the peripheral metabolic effects of insulin in that HFD consumption and obesity are associated with dysregulated IR signaling pathways. Corroborating the idea that the evolutionary function for insulin’s memory enhancing effects might be related to the prandial timing of its secretion, additional prandial factors have been shown to similarly enhance hippocampal-dependent learning and memory (as discussed in the subsequent sections).

## Gut-Derived Vagal Afferents

### System Overview

The vagus nerve (aka, the 10th cranial nerve) is the primary communicator between the gastrointestinal (GI) tract and the brain, delivering energy-status signals from the gut to the brain *via* vagal afferent (sensory) nerves. There are discrete classes of afferent fibers that innervate GI organs to either detect stomach volume or intestinal nutrient contents (Powley and Phillips, 2004; Brookes et al., 2013; Williams et al., 2016). These afferent fibers contain cell bodies in the nodose ganglia that synapse to the CNS. The medial NTS (mNTS) of the hindbrain is an important integrator in gut-to-brain communication, as it is the first brain region to receive vagal-mediated signaling from the GI organs (Grill and Hayes, 2012).

The HPC is a new player in the world of gut-to-brain communication. For example, increased GI signaling by within-meal satiation signals (e.g., gastric distension, intestinal nutrient infusion) activates CBF in hippocampal neurons in rodents (Xu et al., 2008; Min et al., 2011). Additionally, HPC CBF is robustly activated following gastric vagus nerve stimulation (VNS) in obese humans (Wang et al., 2006). However, the mNTS does not directly project to the HPC (Rinaman, 2010; Hsu et al., 2015a). This suggests that the connection between the mNTS and the HPC must be through multi-order neural projection pathways. The locus coeruleus (LC) and medial septum (MS) have been identified as potential brain region relay sites connecting the mNTS to the ventral CA1 HPC (Castle et al., 2005). Consistent with the MS as a potential relay between mNTS and CA1, recent studies demonstrate that stimulation of the vagus nerve induces dHPC theta rhythms *via* MS cholinergic signaling (Broncel et al., 2018). Further, we recently utilized monosynaptic and transsynaptic viral tracing techniques to identify the MS as a relay region connecting the mNTS to glutamatergic neurons in the dorsal CA3 and DG subfields of the dHPC (Suarez et al., 2018). These studies provide support for a multi-order neuroanatomical connection linking GI vagally-mediated signaling to higher-order HPC neural processing.

### Memory Function

While traditionally linked with the mediation of GI-derived satiation/meal termination signaling, the role of the vagus nerve with regards to cognitive function has been a topic of recent interest. Studies from Clark et al. (1995) demonstrate that unilateral cervical VNS and stimulation of vagal afferents with inactivation of efferents (Clark et al., 1998) improves retention of inhibitory-avoidance memory in rats, whereas in humans, VNS enhances retention of recognition memory when stimulation occurred after learning (Clark et al., 1999). Moreover, VNS in rodents improves spatial memory in the Morris water maze as well as inhibitory avoidance learning, whereas elimination of norepinephrine levels in the HPC *via* ICV administration of a neurotoxin selective for noradrenergic neurons reverses these VNS-induced memory enhancements in rats with cerebral ischemia/reperfusion injury (Liu et al., 2016).

While VNS enhances memory retention in a variety of HPC-dependent memory tasks, it is important to consider the physiological role of vagal signaling to memory function. The subdiaphragmatic deafferentation (SDA) procedure, which eliminates all GI vagal sensory signaling to the brain, while leaving 50% of supradiaphragmatic sensory and 50% of vagal motor signaling intact (Norgren and Smith, 1994), has been shown to impair extinction of auditory-cued fear conditioning (Klarer et al., 2014), but did not affect NOR memory or working memory in a non-spatial alternation task (Klarer et al., 2017). Of more direct relevance to HPC-dependent memory, our group has recently shown that subdiaphragmatic vagotomy (SDV), which eliminates both GI sensory and motor signaling below the diaphragm, and nodose ganglia injections of CCK-saporin, a novel surgical method which selectively eliminates ~80% of GI-derived vagal sensory signaling below the diaphragm while preserving 100% of motor signaling, both impair HPC-dependent spatial working and contextual episodic memory function in a Barnes’ maze and novel object in context task. On the other hand, SDV does not affect appetitive learning based on internal energy-state cues and social transmission of food-related cues (Suarez et al., 2018). Thus, these findings suggest that GI-derived vagal sensory signaling, which is engaged during feeding, may promote learning about meal-related visuospatial external environmental cues to remember a food location to facilitate future foraging behavior, while having less impact on memory for nonspatial information surrounding a feeding episode (e.g., social cues and interoceptive circulating energy status cues).

### Neurobiological Mechanisms

Vagal signaling plays an important role in hippocampal neurogenesis and synaptic plasticity. VNS increases BDNF mRNA (Follesa et al., 2007) and activates BDNF receptor TrkB phosphorylation (Furmaga et al., 2012) in rat HPC. Moreover, VNS induces LTP (Zuo et al., 2007), enhances excitatory synaptic transmission (Ura et al., 2013), and increases BrdU (a thymidine analogue incorporated into the DNA of dividing cells; marker of cell proliferation) and doublecortin (a microtubule-associated protein; marker of cell differentiation) immunoreactivity (Biggio et al., 2009) within the DG subfield of the HPC. Importantly, other studies provide physiological relevance of vagus nerve signaling in neurogenesis and synaptic function. Capsaicin-induced sensory vagus nerve injury, which damages non-myelinated vagal afferents, reduced cell proliferation and differentiation in granule cell layer of the HPC in rats (Ronchi et al., 2012). In adult mice, SDV reduces BDNF mRNA in all subregions of the HPC (e.g., DG, CA1, CA3), the number of immature HPC neurons, proliferation in the dHPC, and survival of newly born cells in both dHPC and vHPC (O’Leary et al., 2018). Moreover, rats with either SDV or selective elimination of GI-derived vagal afferent signaling *via* nodose injections of CCK-saporin have significantly reduced BDNF and DCX levels in the dHPC relative to controls, and importantly, these reductions are correlated with the magnitude of HPC-dependent memory impairment (Suarez et al., 2018). Collectively these findings suggest that gut-derived vagal signaling promotes HPC-dependent memory by enhancing neurotrophic and neurogenic pathways, and that the results from O’Leary et al. (2018) discussed above are based on elimination of sensory, and not motor vagal signaling.

### Interactions With Diet and Energy Status

Diet and metabolic factors strongly regulate the sensitivity of vagal afferent neurons to peripheral GI-derived signals. For example, studies have demonstrated significantly reduced c-Fos (a marker of neuronal activation) in the mNTS following a meal in obese rats (Covasa et al., 2000a,b), reduced mechanosensitivity to stomach distension in obese mice (Daly et al., 2011; Kentish et al., 2012), and reduced chemosensitivity to GI hormones that induce satiation in mice maintained on a high-fat diet vs. low-fat-diet (Covasa and Ritter, 1998). This impaired sensitivity of vagal afferent signals that develops in diet-induced obesity may promote reduced satiation and overconsumption of food. In DIO minipigs, chronic VNS decreases food intake, and in a three-choice meal test, reduces sweet food consumption relative to DIO sham-treated animals (Val-Laillet et al., 2010). These findings suggest that VNS may rescue obesity-associated vagally-based deficits in satiation processing. Of relevance to memory function, obese humans with an implantable gastric stimulator (IGS), a device that expands the stomach through electrical stimulation of the vagus nerve, demonstrated 18% higher brain glucose metabolism in the right HPC, measured *via* positron emission tomography (PET), and this was associated with lower emotional eating scores with IGS on relative to off (Wang et al., 2006). These results suggest that obesity-induced blunting of vagal afferent signaling impairs the ability of HPC neural activity to modulate eating behaviors, however, this hypothesis requires further testing.

### Concluding Framework

Within-meal gut-derived vagal sensory signaling may be part of the prandial mechanism that facilitates learning and remembering the physical location of a food source to inform future foraging behavior. These effects likely involve a multi-order neural pathway from the mNTS through the MS to the dHPC that promotes neurogenic and neurotrophic action in HPC neurons. Based on recent results from our group (Suarez et al., 2018), it appears that this pathway is more important for remembering external visuospatial and contextual information compared to social-related cues and circulating energy balance cues related to feeding behavior (Figure 1). Future work is needed to sort out the role of the reinforcer (appetitive vs. aversive vs. passive) in mediating these effects, as well as to examine whether obesity-associate deficits in GI-derived satiation signal processing extend to vagal modulation of memory and cognitive outcomes.

## Glucagon-Like Peptide 1

### System Overview

GLP-1 is produced from the preproglucagon gene. As an incretin hormone, GLP-1 is secreted from the distal intestines and has blood glucose lowering effects (Kreymann et al., 1987; Baggio and Drucker, 2007), attributed primarily to enhanced glucose-stimulated insulin secretion from pancreatic β cells (Mojsov et al., 1987). The primary impetus for GLP-1 secretion from the ileal endocrine L cells is the presence of nutrients in the gut (Baggio and Drucker, 2007). In addition to its incretin actions, GLP-1 signaling also reduces food intake, in part, *via* a reduction in gastric emptying rate (for review, see Shah and Vella, 2014; Drucker, 2018).

In addition to the intestinal GLP-1 secretion, GLP-1 is also released in the brain by the preproglucagon (PPG)-expressing neurons located in the hindbrain mNTS, the caudal medullary reticular formation, and the olfactory bulb (Merchenthaler et al., 1999). Given the short half-life of peripherally-released GLP-1 due to rapid enzymatic degradation by dipeptidyl peptidase-4 (DPP-4), the predominant source of GLP-1 in the brain is thought to be the PPG neurons (Holst, 2007), which project extensively throughout the brain (Gu et al., 2013; Trapp and Cork, 2015). Similarly, GLP-1R is widely expressed throughout the neuraxis (Cork et al., 2015), and several specific nuclei have been identified that are functionally relevant to GLP-1’s food intake reducing effects (for review, see Kanoski et al., 2016).

### Memory Function

Of direct relevance to this review, GLP-1 receptor (GLP-1R) is robustly expressed in the HPC, particularly within the vHPC (Merchenthaler et al., 1999; Cork et al., 2015). GLP-1R activation in the vHPC reduces food intake by reducing meal size and conditioned motivated responding for palatable food *via* downstream connectivity with the medial prefrontal cortex (Hsu et al., 2015a, 2018). In addition to these hypophagic effects, GLP-1 also acts in hippocampal neurons to enhance learning and memory. Early evidence for GLP-1’s role in hippocampal memory function comes from During et al. (2003), who demonstrated that injections of GLP-1 (7–36) amide in the brain (ICV) enhances hippocampal-dependent spatial memory in rodents (During et al., 2003). This report further revealed that GLP1-R KD impairs contextual fear memory in mice and that these impairments are reversed by virogenetic re-expression of the GLP1-R in the HPC (During et al., 2003). These data suggest that GLP-1R signaling in the HPC improves learning and memory in normal healthy animals. However, while GLP-1 crosses the BBB (Kastin et al., 2002), the rapid enzymatic degradation in peripheral circulation by DPP-4 (Holst, 2007) raises the question as to what extent peripheral GLP-1 reaches the brain (and HPC) in meaningful concentrations. Related, GLP-1-releasing PPG neurons in the hindbrain, considered to be the predominant source of endogenous GLP-1 in signaling in the brain, do not project to the HPC, thereby reducing the likelihood of a direct GLP-1 synaptic connection between the NTS and the HPC. Given that GLP-1 immunoreactive terminals directly contact the ependymal cells contacting the cerebral spinal fluid (CSF) in the cerebral ventricles, and that active GLP-1 is present in both the CSF and in hippocampal tissue lysates under physiological conditions, we have previously proposed CSF ventricular “volume transmission” of GLP-1 as a mechanism through which GLP-1 released from PPG neurons reaches HPC neurons and glial cells (Hsu et al., 2015a). Additional support for this biological signaling pathway in the control of feeding behavior was recently provided for the orexigenic neuropeptides, melanin-concentrating hormone and orexin (Noble et al., 2018), thereby further supporting the feasibility of CSF transmission of GLP-1 as a mechanism for communication to hippocampal neurons.

Clinically-relevant evidence supporting a role for GLP-1 in promoting hippocampal-dependent memory comes from studies using peripheral administration of FDA-approved GLP-1-based diabetes and obesity drugs. For example, peripheral treatment with the DPP4 inhibitor sitagliptin, which delays the degradation of GLP-1 and therefore elevates its endogenous half-life, increases markers of hippocampal neurogenesis and improves recognition memory in mice fed a high-fat diet (Gault et al., 2015). Further, chronic peripheral treatment with the long-acting GLP-1 analog, exenatide, significantly improves performance in the spatial radial arm maze in healthy rats (Isacson et al., 2011). Similarly, chronic treatment with a different long-acting GLP-1 analog, liraglutide, improves deficits in spatial memory observed in a rat model of central streptozotocin (STZ)-induced neurotoxicity and diabetes (Palleria et al., 2017), an outcome also observed following exenatide treatment (Chen et al., 2012). In addition to improving hippocampal function in animals with central STZ-induced neurotoxicity, exenatide treatment improves hippocampal-dependent spatial memory in rats made insulin resistant with a high fructose diet (Gad et al., 2016) and in a mouse model of peripheral STZ-induced diabetes (Huang et al., 2012; Gumuslu et al., 2016). Unfortunately, it is not possible from these studies to distinguish the peripheral from the central effects of the GLP-1 analogs, as both exenatide and liraglutide cross the BBB following peripheral administration (Kastin and Akerstrom, 2003; Knudsen et al., 2016). However, ICV injections of GLP-1 attenuate spatial memory impairments in a juvenile model of STZ-induced diabetes (Iwai et al., 2009), suggesting that upregulating central GLP-1R signaling *per se* improves hippocampal-dependent learning and memory in animals with peripheral hyperglycemia and/or reduced insulin production.

It is noteworthy that the food intake-reducing effects of peripherally-delivered GLP-1 analogs (exendin-4 and liraglutide) require vagus nerve-independent BBB transport and direct action on central GLP-1Rs (Kanoski et al., 2011a), suggesting that these GLP-1 analogs may have clinical relevance for memory disorders. Indeed, in rodent models GLP1R agonists have shown promise for reducing the mnemonic deficits associated with AD. In rats, intra-hippocampal injections of GLP1R agonists prevent impairments in spatial memory induced by central injections of amyloid β (Wang et al., 2010; Qi et al., 2016). Similarly, in the amyloid precursor protein/presenilin (APP/PS1) double and APP/PS1/Tau triple transgenic mouse models of AD, liraglutide treatment attenuates spatial memory impairments (McClean et al., 2011; Chen et al., 2017). The memory improvements associated with GLP-1 receptor agonists are not limited to conditions in which amyloid β or hyperphosphorylated tau are present, as GLP-1 receptor agonists are neuroprotective and promote memory retention in senescence accelerated mouse prone 8 (SAMP8) mice, a model of age-related spontaneous AD not associated with amyloid plaques (Hansen et al., 2015).

### Neurobiological Mechanisms

The mechanisms through which GLP-1R activation affects hippocampal-dependent learning and memory are not completely understood, however, evidence suggests that GLP-1R activation increases synaptic plasticity (Gilman et al., 2003; Abbas et al., 2009; Gault et al., 2010; Han et al., 2013; Cai et al., 2014) and increases neurite outgrowth (Perry et al., 2002). GLP-1R agonism has also been shown to affect gene expression levels of the neurotrophic and neuroplasticity factor BDNF and its receptor TrkB (Lennox et al., 2014; Gumuslu et al., 2016). However, other studies found no effect of GLP1R agonist liraglutide treatment on expression levels of NTRK2, BDNF, or synaptophysin, but nevertheless observed an increase in hippocampal LTP in the CA1 region which was associated with elevated levels of the mammalian achaete-scute homologue 1 [Mash1; a marker for neurogenesis (Isacson et al., 2011; Porter et al., 2013)]. GLP-1R agonists also increase neurogenesis in the HPC, evidenced by increased expression of the neurogenic marker doublecortin, elevated BrdU incorporation, and an increase in neuronal number in the HPC (Isacson et al., 2011; Hansen et al., 2015). Thus, taken together GLP-1 may improve HPC-dependent learning and memory by increasing neuronal plasticity, neurogenesis, and/or both.

### Interactions With Diet and Energy Status

Unlike previous systems discussed above, results on whether dietary and metabolic factors regulate GLP-1 sensitivity are mixed. Postprandial GLP-1 release is attenuated in obese individuals (Näslund et al., 1998) and higher levels of GLP-1 appear to be required to produce anorectic effects and terminate a meal (Näslund et al., 1999), suggesting obesity-induced GLP-1 insensitivity, or “resistance” with regards to feeding outcomes. Consistent with these effects, rats maintained on a HFD demonstrate attenuated satiation effects following GLP-1 receptor activation (Williams et al., 2011). Of relevance to memory outcomes, peripheral administration of GLP-1 receptor agonists [e.g., Liraglutide (Val8)GLP-1(GluPAL), Exendin-4] in DIO mice rescues HPC LTP and normalizes object recognition memory to performance levels similar to lean control mice (Gault et al., 2010; Porter et al., 2010; Lennox et al., 2014). Additionally, oral administration of Sitagliptin (a DPP-4 inhibitor that elevates GLP-1 concentrations) reverses object recognition memory deficits and elevates HPC doublecortin expression in HFD-maintained mice (Gault et al., 2015).

In contrast with these findings, however, human studies demonstrate limited or no evidence of obesity-associated GLP-1 resistance following chronic pharmacological GLP1 analog treatment. For example, exenatide treatment produces significantly greater weight loss in extremely obese individuals relative to that observed in overweight or lean individuals (Blonde et al., 2006). Moreover, intravenous administration of exenatide reduces blood-oxygen-level dependent responses in a memory-related brain regions (including the right HPC) in response to high-calorie food pictures in obese subjects but not in lean subjects (Eldor et al., 2016). Overall the influence of dietary and metabolic factors on GLP-1-based effects on incretin signaling, feeding behavior, and memory outcomes is thus far poorly understood.

### Concluding Framework

As highlighted throughout this review, there may be an evolutionary benefit in remembering the details surrounding a meal, such as remembering where the food source comes from, as well as whether the meal rewarding/nutritive or did it cause malaise. Given the role of the HPC in memory function related to spatial and temporal events, contextual elements associated with reward or aversive stimuli (Behrendt, 2013), or physiological energy status cues (reviewed in Kanoski and Grill, 2017), it seems logical that the time during and immediately following food consumption is a key time for the HPC contribute to encode memory. While the GLP-1 system has similar effects to insulin on hippocampal function, the fact that there are direct effects of GLP-1 in the brain independent of the incretin effects of GLP-1 in the periphery (During et al., 2003; Iwai et al., 2009) suggests that these two systems have independent effects on memory function. This redundancy supports a framework in which enhanced memory function during and surrounding meal consumption involves multiple energy balance relevant systems that have different temporal secretion dynamics, as well systems that have opposing effects on food intake (Figure 1). Consistent with data discussed above indicating that these systems are modulated by energy status, now we discuss a system, leptin, that is a key signal for chronic energy status.

## Leptin

### System Overview

Leptin is a 16 kD hormone synthesized and released into the blood primarily by white adipose tissue (Zhang et al., 1994). Circulating plasma leptin levels are positively correlated with fat mass and fluctuate with nutrient status (Frederich et al., 1995; Maffei et al., 1995), with low plasma levels during nutrient deficiency and high levels postprandially and in obese states (Dalamaga et al., 2013). Additionally, there is a stomach-derived source of leptin, which is secreted during a meal and absorbed from the small intestine into the blood stream (Cammisotto and Bendayan, 2012). Leptin exerts its main effect on long-term regulation of energy homeostasis by binding to the long isoform of leptin receptor (LepRb) expressed in peripheral tissues and the brain. Leptin acts as an energy status signal to reduce food intake and body weight during times of nutritional abundance, whereas mutation of leptin or LepRb results in severe hyperphagia, decreased energy expenditure, and early onset obesity in humans and rodents (Tartaglia et al., 1995; Chua et al., 1996; Montague et al., 1997; Farooqi et al., 1999; Kelesidis et al., 2010). Leptin crosses the BBB *via* a saturable transporter system (Banks et al., 1996) and binds to LepRbs expressed in the hypothalamus, caudal brainstem, and elsewhere to regulate energy balance control (Friedman and Halaas, 1998; Bates and Myers, 2003; Myers et al., 2009; Hayes et al., 2010; Kanoski et al., 2012). Recent findings have also highlighted the physiological relevance of LepRb signaling in higher-order (limbic, cortical) brain regions, including the lateral hypothalamus (Leinninger et al., 2009, 2011; Goforth et al., 2014; Laque et al., 2015), ventral tegmental area (Fulton et al., 2006; Hommel et al., 2006; Mietlicki-Baase et al., 2015), and of direct relevance to this review, the CA1, CA3, and DG subregions of the HPC (Huang et al., 1996; Couce et al., 1997; Shioda et al., 1998; Burguera et al., 2000b; Ur et al., 2002).

### Memory Function

In addition to its more widely studied role in energy balance, leptin also promotes memory function, particularly through its action in hippocampal neurons. For example, intravenous and peripheral administration of leptin enhances performance in HPC-dependent spatial and contextual memory tasks in rodents (e.g., Morris water maze and passive avoidance task, respectively; Oomura et al., 2006), as well as a novel place recognition task that measures contextual episodic memory (Malekizadeh et al., 2017). In humans, leptin deficiency is associated with impaired verbal memory function, which is rescued following subcutaneous leptin administration (Paz-Filho et al., 2008). Within the brain, direct dHPC leptin administration improves HPC-dependent memory retention in mice in a T-maze foot shock avoidance and step-down avoidance tasks (Farr et al., 2006). Collectively, these behavioral studies indicate that leptin signaling improves HPC-dependent memory function associated with passive reinforcement tasks and memory tasks based on escape/aversive reinforcement.

Leptin’s pharmacological effects on memory may be particularly influenced by the nature of the reinforcement. For example, the studies mentioned above reveal that leptin, administered either peripherally or directly to the dHPC, improves memory function for spatial and contextual-based tasks that involve passive or aversive reinforcement. In contrast, leptin administration in the vHPC *impairs* memory consolidation for the spatial location of food, whereas dHPC administration had no effect (Kanoski et al., 2011b). Consistent with these findings, vHPC lesions, which eliminate leptin signaling in the vHPC, enhance memory for an association between environmental cues and food reward (Ferbinteanu and McDonald, 2001). It could be that increased leptin signaling in the vHPC, associated with energy surplus, reduces the ability of environmental cues to elicit memory for food-related information in favor of remembering non-food-related cues. Whether this putative reinforcement-dependent role for leptin in memory function is differentially mediated by dorsal vs. ventral hippocampal neurons requires further investigation.

### Neurobiological Mechanisms

At the neuronal level, leptin acts on HPC neurons to promote synaptic function, in part, by protecting HPC neurons from apoptosis (Guo et al., 2008), by promoting proliferation of HPC progenitor cells (Garza et al., 2008), and by increasing dendritic spine formation (O’Malley et al., 2007; Stranahan et al., 2009; Dhar et al., 2014a,b). LepRb deficient rodents (e.g., Zucker fa/fa rats and db/db mice) demonstrate impaired LTP and LDT in HPC CA1 synapses, as well as impaired spatial memory performance in the Morris water maze (Li et al., 2002). These changes in LTP and LTD are mediated, in part, by leptin-dependent regulation of N-Methyl-D-aspartate (NMDA) receptor activation (Shanley et al., 2001; Durakoglugil et al., 2005), as well as through activation of Akt/PI3K and ERK/MAPK intracellular signaling pathways (Niswender et al., 2001; Irving et al., 2006; Weng et al., 2007; Garza et al., 2008; Guo et al., 2008). PI3K signaling, in particular, is implicated in leptin-dependent induction and maintenance of LTP, LTD, and NMDA receptors in the HPC (Shanley et al., 2001; Durakoglugil et al., 2005) *via* stimulation of calcium/calmodulin-dependent protein kinase II (CaMKII) activity (Li et al., 2002; Oomura et al., 2006). In addition to enhancing synaptic plasticity, leptin induces morphological changes by forming dendritic protrusions through activation of TrpC signaling, which is important for BDNF-induced spine formation in the HPC (Li et al., 2010), *via* activation of CaMK signaling (Dhar et al., 2014a), and by increasing dendritic spine density of HPC synapses through activation of NMDA receptors that is mediated by MAPK/ERK signaling pathway (O’Malley et al., 2007).

While leptin plays an important role in enhancing neuronal plasticity and memory function, studies demonstrate that variations in nutritional status can alter these leptin-mediated effects. Low serum levels of leptin have been linked to severe malnutrition (Caro et al., 1996; Pinos et al., 2007; Amirkalali et al., 2010), acceleration of brain aging, and higher risk to develop AD (Power et al., 2001; Lieb et al., 2009; Fadel et al., 2013). Further, serum leptin levels are positively correlated with HPC gray matter volume (Lieb et al., 2009; Narita et al., 2009) and MCI patients demonstrate lower serum leptin levels independent of body fat, as well as reduced memory performance on an auditory verbal learning test and decreased volume and microstructural integrity of HPC subfields relative to healthy controls (Witte et al., 2016). Direct administration of leptin to HPC neurons, either *in vitro* or *in vivo*, facilitates LTP induction, whereas smaller and larger doses of leptin actually inhibit LTP (Wayner et al., 2004). These findings suggest that a specific concentration of leptin when optimal energy reserves are met may enhance synaptic plasticity, with opposite effects observed in the presence of either insufficient or excess energy reserves. Moreover, leptin rescues the increased neuroapoptosis and impaired cognitive function in the Morris water maze *via* regulation of 5′-AMP-activated protein kinase (AMPK) activity that occurs under severe dietary restriction (Dagon et al., 2005). Therefore, nutrient deficiency (associated with low circulating leptin levels) impairs HPC integrity and cognitive function, further supporting the notion that leptin’s role as an energy-status signal may extend to modulating HPC-dependent spatial and other memory processes.

### Interactions With Diet and Energy Status

Obese rodents and humans demonstrate leptin resistance and peripheral hyperleptinemia, in part, due to reduced leptin transport across the BBB and reduced ability of CNS leptin to engage intracellular signaling pathways downstream of LepRb binding (Montague et al., 1997; Strobel et al., 1998; Banks et al., 1999; Farooqi et al., 1999; Burguera et al., 2000a; El-Haschimi et al., 2000; Banks, 2001; Munzberg et al., 2005). Considering that obesity is associated with impaired cognitive function (Elias et al., 2005; Cournot et al., 2006; Wolf et al., 2007), leptin resistance may play a role in HPC-dependent memory impairment associated with obesity (see Van Doorn et al., 2017 for further review on this topic). Related, rats maintained on a high fructose and HFD develop hyperleptinemia, lower central leptin levels, and impaired performance on the Morris water maze task (Lin et al., 2017). These effects could be due to elevated stearoyl-CoA desaturase-1 (SCD-1) expression in the brain, an enzyme involved in fatty acid metabolism with expression correlated with obesity and suppressed by leptin (Biddinger et al., 2006). Moreover, mice maintained on a HFD demonstrate impaired spatial memory in a novel location recognition (NLR) task with desensitization of PI3K/Akt signaling pathway coupled to HPC LepRbs (Valladolid-Acebes et al., 2013).

Excessive caloric consumption may interfere with leptin’s capacity to promote memory function through interactions with BDNF, a neurotrophin that promotes plasticity and is reduced in the HPC following HFD maintenance in rodents (Molteni et al., 2002; Kanoski et al., 2007; Noble et al., 2014). For example, CNS leptin increases BDNF expression in the HPC, and this effect is diminished with HFD maintenance (Yamada et al., 2011; Kanoski et al., 2014). Consistent with this framework, obese rats (induced *via* lentivirus-mediated downregulation of hypothalamic IRs) demonstrate hyperleptinemia and impaired LTP in CA1 HPC (Grillo et al., 2011a), as well as deficits in HPC synaptic plasticity, reduction in HPC dendritic spine density, and reduced c-Fos expression in the CA1 subregion of the HPC following fear conditioning tasks (Grillo et al., 2011b). Rats on a high-calorie diet also demonstrate reduced HPC LTP at CA1 synapses and deficits in spatial memory function through BDNF-mediated effects on dendritic spine density (Stranahan et al., 2008).

### Concluding Framework

Results discussed above indicate that the HPC receives and responds to leptin-induced energy status signaling to influence memory function. Interestingly, data support a U-shaped mechanism whereby low and high concentrations of leptin present in malnutrition and obesity, respectively, negatively impact HPC synaptic plasticity and cognitive function. Therefore, when energy reserves are met and leptin levels are optimal to maintain energy homeostasis, the HPC appears to be able to integrate information about healthy energy status to promote spatial and contextual memory, which may be relevant to efficiently learning the location of food and other episodic elements surrounding feeding (Figure 1). Future research is needed to determine whether HPC leptin signaling differentially promotes memory based on food vs. nonfood reinforcement, and related, whether these effects are dependent on HPC subregion (dorsal vs. ventral), as well as overall energy status and circulating leptin levels.

## Summary

Eating is paramount in the hierarchy of mammalian physiological and psychological needs. The literature reviewed herein highlights that the neurobiological control of learning and memory is strongly linked with signaling from biological peripherally-derived systems that fluctuate around and strongly influence the consumption of a meal (Figure 1). Preprandial (ghrelin) and prandial/immediate postprandial signals, including insulin, GLP-1, and gut-derived vagal nerve activation, improve HPC-dependent contextual, episodic, and spatial memory. Similarly, the adipocyte-derived hormone leptin, an overall energy status marker, has similar beneficial effects on HPC neural and mnemonic outcomes. While beyond the scope of this review, it is also the case that various CNS-derived neuropeptide systems that potently influence appetite and feeding enhance hippocampal-dependent memory *via* similar neuronal mechanisms as the peripheral-derived systems discussed herein. For example, the hypothalamic-derived feeding-related neuropeptides, orexin (aka, hypocretin; Yang et al., 2013; Mavanji et al., 2017), oxytocin (Lee et al., 2015), melanin-concentrating hormone (Monzon et al., 1999; Adamantidis et al., 2005), and neurotensin (Ohinata et al., 2007; Zhang et al., 2016), have all been established to promote HPC memory function and plasticity in rodent models. Collectively, these peripheral- and central-derived biological systems represent a diverse array of neurobiological pathways that bi-directionally influence feeding behavior, yet generally, have common promoting/enhancing effects on HPC function, with some exceptions (e.g., based on pharmacological dose effects, nature of reinforcement, etc.).

The common neurobiological mechanisms through which these diverse systems improve memory function include (but are not limited to) enhanced neuroplasticity and neurogenesis in hippocampal neurons. Based on evidence that multiple signals elevate BDNF-TrkB signaling, which is important for both synaptic plasticity and hippocampal neurogenesis, it is possible that this neurotrophic pathway may be downstream of both preprandial and prandial/postprandial feeding-related biological signals, and upstream of neuroplasticity, neurogenic, and mnemonic outcomes. Though this hypothesis remains to be tested, it is possible that surrounding the event of a meal, energy balance-related signaling factors promote a temporary rise in BDNF/TrkB signaling which primes neuronal systems for learning about the critical elements of the meal. Importantly, dietary and metabolic factors appear to potently regulate the capacity of these systems to promote these neurobiological mechanisms and enhance memories of a meal, and generally-speaking, the memory-promoting capacity of these systems are reduced with obesity and HFD maintenance.

This review highlights functional/behavioral data from both humans and mechanistic experimental rodent model experiments revealing that hippocampal-dependent memory function is enhanced by biological systems that show sharp and dynamic fluctuations surrounding mealtime. Such mnemonic enhancement likely provides an important evolutionary advantage. For example, the ability to recall the details surrounding a nutritive meal, including the physical location of the food, how to procure and prepare it, and what external episodic factors modulate the availability and nutritive quality of the food, may serve to increase the success and efficiency of future foraging and feeding behavior. The reviewed literature suggests that these meal-related systems also enhance the capacity for memory for events not directly related to feeding behavior, including those that involve passive or aversive reinforcement. Whether these effects are a by-product of systems that are engineered to promote feeding-related memories, and related, whether the nature of the reinforcer (food vs. nonfood, hedonic vs. homeostatic, appetitive vs. aversive) is a critical factor in the ability of these systems to promote memory function, are important areas for future research.

## Author Contributions

All authors made comparable scholarship, creative, and writing contributions to this review article.

## Conflict of Interest Statement

The authors declare that the research was conducted in the absence of any commercial or financial relationships that could be construed as a potential conflict of interest.
